# Progress in Medicine

**Published:** 1895-01-26

**Authors:** 


### PROGRESS IN MEDICINE.

### DISEASES OF CHILDREN.

(*Continued from page* 276.)

**Diseases of the Long Bones.**—In recent papers some important additions have been made to our knowledge of the disease, or diseases, variously described under the names, "periosteal abscess or acute necrosis," "acute epiphysitis," "acute epiphyseal necrosis," "acute, malignant, or infective periostitis," "acute, infective, or septic osteo-myelitis," "septic osteitis," 'para-epiphysitis," &c. Mr. T. Pickering Pick[1] points out that many of these names are misleading, because the disease referred to does not, in the first instance at least, involve the epiphysis, but commences in the growing tissue at the end of the diaphysis, on the surface of the epiphyseal cartilage remote from the joint. In this situation there is a quantity of jelly-like embryonic marrow, composed of branched cells and osteoblasts, supplied with numerous large blood-vessels, and continuous with the osteogenic layer of the periosteum. In this tissue the life of the limbs reaches its highest point of functional activity, and, therefore, it is liable to proportionally increased risk of harm from injury or disease. Another situation of similar functional activity, and similar liability to disease, is the epiphysis itself. In the growing medulla of young bone, and in the deep vascular layer of periosteum, septic material is apt to start those inflammations which we term acute osteo-myelitis, and acute diffuse periostitis, and similarly the poisons of syphilis and tubercle are apt to flourish here in the young. Affections of the ends of the long bones he classifies under the three headings, acute, sub-acute, and chronic. The acute variety is, in the majority of cases, the result of an injury, and occurs in children under one year who are in a delicate state of health. He discusses the question as to whether this is a pyæmic affection, and decides that it is not, for the following reasons: (1) That a larger number of cases are successfully treated than could be expected if the disease were a true pyæmia; (2) that recovery and repair of tissues are so rapid; (3) that the disease is confined to one or more bones, and other tissues are not involved, and

(4) that the suppurative arthritis, which is so often present, is not pyæmic in character, but is due to the bursting of the abscess, which has formed in the bone, into the cavity of the joint. At the same time, while denying a pyæmic origin to these cases, he admits that the disease may lead to pyæmia secondarily, as pyogenic cocci are always present. This form of septic osteomyelitis affects most commonly the lower end of the femur, the upper end of the femur, and the upper end of the humerus. Pus forms rapidly, and soon penetrates into the neighbouring joint, or it may be diffused under the periosteum. The sub-acute form is usually confined to one bone, and the joint is rarely involved. The chronic variety is associated with tuberculosis or syphilis, and here again he lays stress on the fact that the disease originates most frequently in the growing tissue at the end of the diaphysis. When the upper end of the femur is affected, the disease often involves the joint secondarily, and this represents the sequence of events in many cases of hip joint disease. As regards the osteal hæmorrhages of infantile scurvy, he states that bleeding commences in the vascular and growing tissue of the ends of the shafts, and that from this point the blood travels to the surface of the bone, and thence along under the periosteum, which is gradually detached from the shaft.

Dr. C. F. Marshall[2] has recorded two cases of "suppurative epiphysitis of infants," which were under the care of Mr. Owen and Mr. Pitts. In both children the primary cause was found in cutaneous sores, which led to secondary lesions in the long bones and very rapidly to a fatal termination. The ribs were affected in each case, and in one patient both clavicles were involved—a somewhat unusual condition.

In a lecture on "Septic Osteitis in Childhood" Mr. Edmund Owen[3] points out that this affection usually assumes an acute and fulminating character, and may terminate fatally before a diagnosis has been made. More rarely it is of a chronic type. The primary mischief consists of a septic inflammation in the young osseous tissue at the end of the diaphysis, which spreads rapidly through the bone and marrow. Early treatment is of the first importance, and too often a mistaken diagnosis of rheumatism has led to the loss of the limb affected, if not of the patient's life. In distinguishing septic osteitis from articular rheumatism, he mentions the following points: (1) the heat, swelling, pain, and redness present in osteitis are close to, but do not involve the joint; (2) a distinct thickening around the bone can always be detected, and (3) the treatment by salicin and its compounds has no effect. He recommends early and thorough operative measures. An incision should be made down to the bone, and any pus present under the periosteum evacuated. If the junction cartilage is affected it must be scraped and irrigated. If no pus is found beneath the periosteum, the diaphysis of the bone should be trephined in several places, the intervening pieces of bone cut away, the suppurating marrow scraped out, and the cavity swabbed with chloride of zinc solution.

With reference to this subject, Dr. Colcott Fox[4] points out that the similarity to rheumatism is increased in some cases by the presence of an urticarial eruption. He describes a case—that of a girl aged thirteen years—who presented symptoms of septic osteo-myelitis, and who developed purplish-red patches over the trunk and limbs. At the necropsy, the lower two-thirds of the left femur were found denuded of periosteum and surrounded with pus, while many secondary abscesses were present in the joints and viscera.

**Hereditary Syphilis.**—Professor Fournier[5] divides the evidences of hereditary syphilis into five groups: (1) Signs presented by the general external appearance; (2) cicatrices of the skin; (3) lesions of the skeleton; (4) condition of the testicle; and (5) the so-called "triad of Hutchinson." If the scars are circular or serpigenous in outline, or extensive, or situated around the mouth and nose, or in the lumbar, gluteal, and crural regions, a syphilitic origin is probable. The joint lesions are of two kinds, hydropathies and arthropathies with deformities. The testicles are usually small, either from non-development or because of a fibro-sclerotic degeneration leading to atrophy.

An interesting example of hydrarthritis in a boy aged fourteen, the subject of hereditary syphilis, is recorded by Mr. D'Arcy Power.[6] The knees and elbows were affected, there being considerable enlargement and thickening of the synovial membrane. The other symptoms present were extensive ulceration and scarring of the skin, and laryngitis. Under anti-syphilitic treatment the boy rapidly recovered. Mr C. A. Ballance[7] relates a case of symmetrical synovitis of both knees, associated with hereditary syphilis. The patient, a boy aged six years, had suffered in infancy from convulsions followed by right hemiplegia. The signs of syphilis were not very well marked, but as both knee joints contained fluid, syphilis was diagnosed, and the boy treated accordingly with the best results.

**Hernia and Herniotomy.**—Dr. Southgate[8] has analysed seventy-one cases of hernia with a view to determining the etiology. He admits as factors the influence of heredity and race, anatomical defects (such as a long mesentery, or imperfectly formed muscles in the abdominal wall), but lays special emphasis on rickets. Fifty-two of his cases were under two years of age, and 29·5 per cent. of the total number showed well marked signs of rickets. Taking these facts into consideration, he is inclined to regard rickets as causally related to hernia, both from the general weakness of the system which is present, and from the local weakening of the abdominal wall which is thinned out over the distended intestines. Dr. S. E. Milliken[9] describes a case in which he did a radical cure for inguinal hernia in a child of five years. He operated on both sides, and advises this treatment, by Bassini's method, when mechanical measures cannot be effectively carried out. Dr. Carl Stern[10] has collected and analysed fifty-one cases of herniotomy for strangulated inguinal hernia in children under four years of age. The mortality was 22·6 per cent. Statistics show that the mortality in this operation has been reduced from 33 to 21 per cent. by the adoption of antiseptic measures.

**Craniotomy in Microcephalus.**—In an admirable address entitled "Non Nocere," Dr. Jacobi[11] discusses some forms of congenital constipation, the local treatment of diphtheria, and the question of surgical interference in cases of microcephalus. He denies that premature ossification is the usual accompaniment of microcephalus, and maintains that disease of the brain or its membranes is the real cause of the small size of the head. The histories of thirty-three patients on whom forty-one operations were performed have been obtained. There were fourteen deaths and nineteen recoveries. The benefits observed in those cases which survived are of such a slight nature as fully to justify Dr. Jacobi's statement that "the operation is not promising of good to mankind." Dr. A. Thomson[12] describes the post-mortem appearances in two cases of microcephalus, the subjects of which had survived to adult life. In both cases there seemed to be no doubt that the primary defect was in the brain itself, and that the small size of the skull was a secondary lesion. The microcephalic brain is predisposed to disease, the condition is often hereditary, and the subjects of this affection usually succumb early to pulmonary phthisis.

**Parotitis and Orchitis.**—Dr. Catrin[13] says that the orchitic complications of mumps are of two kinds, firstly, testicular congestion, and secondly, orchitis. The former is not serious, and never ends in atrophy. The latter develops from four to eight days after the onset of an attack of mumps, is accompanied by pain and pyrexia, originates in the epididymis, and may be confined to that part or affect the whole testicle. Absolute rest in bed during an attack of mumps is a measure which is invariably successful in preventing this complication.

An illustrative case is published by Dr. W. G. Creswell.[14] A lad aged seventeen complained of pain in the right testicle on the fourth day of an attack of double parotitis. The following day the temperature was 105 deg., his pupils were dilated, and vomiting and delirium were present. The right testicle was red, hot, and swollen. Under suitable treatment the patient made a complete recovery in ten days.

In an epidemic of mumps affecting forty-eight soldiers in garrison, Dr. Martin[15] states that orchitis was present in nine cases, or 18 per cent. He does not look on orchitis as a complication, but as representing the second degree of the poison of mumps. Pyrexia was always present when the testicle was affected.

**The Treatment of Empyema.**—Mr. George Heaton[16] gives the statistics of seventy cases of empyema treated at the Children's Hospital, Birmingham. In two cases aspiration was successfully employed, and the other sixty-eight cases were treated by incision and drainage. There were in all nine deaths, or a mortality of 12·8 per cent., but as in three of these cases pericarditis, and in one nephritis was the cause of death, the mortality is reduced to 7·6 per cent. The writer does not approve of aspiration except as a temporary measure, and recommends free incision and drainage as the best method of treatment. He does not aim at a complete evacuation of the abscess at the time of operation, but a slow removal of the pus into an absorbent dressing, so as to avoid the dangers of collapse or pulmonary hæmorrhage. In the after treatment stress is laid on the benefit derived from the regular employment of chest expanding exercises. Dr. A. E. Morison[17] has operated on thirty-four cases of empyema with two deaths, or a mortality of 5·8 per cent. He considers aspiration disappointing as a curative agent, and recommends incision and drainage. Mr. T. P. Wightman[18] gives some statistics regarding 124 cases of empyema treated at the Infirmary for Children, Liverpool. The number of deaths was twenty-nine, a result which does not afford much support for the line of treatment adopted—namely, incision and drainage.

Dr. Batten[19] writes in support of the treatment by resection of a rib and drainage, and gives the statistics for one year at the Hospital for Sick Children, London. There were forty-eight cases in all, with eight deaths, but as three of these died from causes quite independent of the empyema, the mortality is reduced to 5 or 11 per cent. This method of treatment has the support of Dr. Aufrecht,[20] of Magdeburg, who advises in addition irrigation of the abscess cavity with a 2 per cent. carbolic solution. He has treated thirty-six cases with one death, and the average duration of treatment was forty-eight days.

1 Lancet I., 1894, pp. 1,543, 1,601. 2 Lancet I., 1894, p. 1,131. 3 Lancet I., 1894, p. 1,285. 4 Lancet II., 1894, p. 255. 5 La Presse Med., Ap. 24th and May 5th, 1894, and Med. Chron., July, 1894 6 Lancet I., 1894, p. 1,618. 7 Clin. Jour., May 16th, 1894, p. 49. 8 Arch. of Ped., Ap., 1894, and Inter. Med. Mag., Aug., 1894. 9 Med. Rec., July 14, 1894, p. 39. 10 Centralbl. für Chirurg., No. 19, 1894, p. 425, and Annals of Surg., Sept., 1894. 11 N.Y. Med. Rec., May 19th, 1894. 12 Jour. of Anat. and Phys., July, 1894, and Ep.B.M.J., II., 1894, p. 13. 13 Med. Week, Feb. 23rd, 1894, p. 91. 14 Birm. Med. Rec., Ap., 1894. 15 Rev. de Med., March, 1894, and Ep., B.M.J., Ap., 1894. 16 Birm. Med. Rev., Sep., 1894. 17 Lancet, Sep. 29th 1894. 18 Lancet I., 1894, p. 1,128. 19 Lancet I., 1894, p. 1,368. 20 Arch. für Klin. Med. Band lii., 1893, p. 1, and Int. Med. Mag., Jany., 1894.

